# Common Mycorrhizae Network: A Review of the Theories and Mechanisms Behind Underground Interactions

**DOI:** 10.3389/ffunb.2021.735299

**Published:** 2021-09-30

**Authors:** Aline Fernandes Figueiredo, Jens Boy, Georg Guggenberger

**Affiliations:** Institute of Soil Science, Leibniz Universität Hannover, Hannover, Germany

**Keywords:** resources allocation, plant fitness, mycelium connections, connected plants, direct pathway, indirect pathway

## Abstract

Most terrestrial plants establish symbiotic associations with mycorrhizal fungi for accessing essential plant nutrients. Mycorrhizal fungi have been frequently reported to interconnect plants via a common mycelial network (CMN), in which nutrients and signaling compounds can be exchanged between the connected plants. Several studies have been performed to demonstrate the potential effects of the CMN mediating resource transfer and its importance for plant fitness. Due to several contrasting results, different theories have been developed to predict benefits or disadvantages for host plants involved in the network and how it might affect plant communities. However, the importance of the mycelium connections for resources translocation compared to other indirect pathways, such as leakage of fungi hyphae and subsequent uptake by neighboring plant roots, is hard to distinguish and quantify. If resources can be translocated via mycelial connections in significant amounts that could affect plant fitness, it would represent an important tactic for plants co-existence and it could shape community composition and dynamics. Here, we report and critically discuss the most recent findings on studies aiming to evaluate and quantify resources translocation between plants sharing a CMN and predict the pattern that drives the movement of such resources into the CMN. We aim to point gaps and define open questions to guide upcoming studies in the area for a prospect better understanding of possible plant-to-plant interactions via CMN and its effect in shaping plants communities. We also propose new experiment set-ups and technologies that could be used to improve previous experiments. For example, the use of mutant lines plants with manipulation of genes involved in the symbiotic associations, coupled with labeling techniques to track resources translocation between connected plants, could provide a more accurate idea about resource allocation and plant physiological responses that are truly accountable to CMN.

## Mycorrhiza Network: Theoretical Background

Mutualistic associations between mycorrhizal fungi and plants are well-known. Within the diverse mycorrihza types, the arbuscular mycorrhizae (AM), from the phylum Glomeromycota, is one of the most common, ancient and widespread, associating with around 80% of all land plant species (Schüßler and Walker, 2011). This fungi type is more predominant in warm climates and species rich ecosystems, such as tropical forests. The second most common fungi type in nature is the ectomycorrhizal (EM) fungi. Although a lower number of plant species have been found to form symbiosis with EM, in comparison to AM, the hosts of EM tend to be widely dispersed, abundant and dominant members of their groups (Brundrett, 2009; Teste et al., 2020). Different from AM, EM fungi are mainly found in colder regions and ecosystems, where less host species are present, e.g., temperate and boreal forests (Brundrett, 2009; Gorzelak et al., 2015). AM and EM networks are assumed to differ in their structure, but both affect plant responses, such as growth, photosynthesis rate, nutrition, survival, and others (Gorzelak et al., 2015). Besides, AM and EM fungi species are frequently found co-existing in the same ecosystem. Some exceptional plants are even able to host both types of fungi in its roots, although the proportion of the association with each may differ along plant's life (Gorzelak et al., 2015).

Mycorrhizae fungi are widely recognized to improve plant nutrition by being able to access soil spaces and nutrient sources inaccessible for roots (Smith and Read, 2010, Wipf et al., 2019; Andrino et al., 2021). The great majority of mycorrhizae fungi are not host specific, being that a single mycorrhizae fungi specie is able to colonize a wide range of plant species. Once a fungi colonize the host plant, its mycelium is able to grow over large distances in the soil and may reach and colonize the roots of multiple neighboring plants, from the same or different species (Van Der Heijden and Horton, 2009). Therefore, plants sharing the same host fungi are reported to become interconnected by the so-called common mycorrhiza network (CMN) (Heaton et al., 2012; Rhodes, 2017; Wipf et al., 2019). Connectivity are therefore likely to occur between plants able to associate with the same fungi species.

As ecosystems are usually dominated by mycorrhizal plants, including most temperate and tropical grasslands as well as boreal, temperate and tropical forests (Read, 1991; Van Der Heijden, 2016), abundant and extensive mycorrhizal fungal networks are formed (Wipf et al., 2019). It is believed that plant species can interact and communicate via these CMNs (Gorzelak et al., 2015; Pickles et al., 2017; He et al., 2019). This may affect survival and behavior of connected plants as well as competitive and cooperative patterns, consequently influencing plant diversity at local and regional scales (Deslippe and Simard, 2011; Simard et al., 2012; Bücking et al., 2016). Among the reported effects of such connectivity are the improvement of seedling establishment (Bingham and Simard, 2011; Seiwa et al., 2020), impact on plant and microorganism community compositions (Meng et al., 2015; Teste et al., 2015; Kadowaki et al., 2018), induction of plant defense responses (Babikova et al., 2013; Song et al., 2014), plant communication through a variety of phytohormones such as jasmonic acid, methyl jasmonate and zeatin riboside (Song et al., 2010), and nutrient exchange, which may play a pivotal role for interplant nutrition (Bücking et al., 2016; He et al., 2019; Fang et al., 2021).

In the review made by Van Der Heijden and Horton (2009) it is stated that CMN can be compared either to “socialist” or “capitalist” systems, or even to a “superorganism.” For the “socialist” behavior, individuals are able to have equal opportunities and resources are distributed more evenly providing benefits for all connected plants. For the “capitalist” network, mycorrhizal would be privately controlled for the profit of certain group of plants, increasing therefore competition between connected plants. If network behaves as a “superorganism,” fungal species in the network are considered redundant physical extensions of the roots, which might translocate nutrients freely between plants. Therefore, the mode of interplant connection might have evolutionary consequences of CMN by substantially defining the community ecology of a site, leading to ecosystem-wide impacts (Gorzelak et al., 2015). This depends largely on which of these responses are predominant (“socialist,” “capitalist,” or simple physical extensions) in the moment plants are connected; together with the question whether these responses may change if plants from the same or from different species are connected.

In face of all the possible effects of CMN on plant interactions, many different theories have been raised with the intention to predict how mutual association and co-existence of species in the system is stabilized. By one hand, we have the biological market theory, for example, which is based on the assumption that fungi might recognized the best plant partner and re-allocate nutrient accordingly to its carbon (C) gain. On the other hand, we have the source-sink theory in which resource would move in a concentration gradient. This could lead resources to be distributed more equally among partner involved in the network, which is the opposite of what is expected if the biological market is driven resource allocations. Both theories will be more detailed discussed in the following sections. Nevertheless, benefits and disadvantages from the interactions between connected plants are hard to distinguish in nature, once most of the plants are colonized simultaneously by multiple fungal species, each one with its own cost–benefit. In addition, in natural ecosystems, not only mutualistic interaction between connected mycorrhizal plants takes place, but networks may also include commensalistic and even antagonistic interactions (Toju et al., 2013). Therefore, some plant species might benefit from CMN more than others, depending on the fungi and plants involved in the association. It is important to note that, even if plants would be connected mainly by a single mycorrhiza type, i.e., AM fungi, variations in the functional properties and temporal patterns of different strains can also be observed (Kiers et al., 2011). This adds further complexity to the potential mechanisms by which such network would determine plant community composition and productivity through their facilitative and antagonistic effects on plants (Wagg et al., 2015). Therefore, predicting ecosystem dynamics of connected plants is still a huge challenge.

Due to the high complexity to discriminate effects of CMN in natural ecosystem, the majority of studies aimed to evaluate the influence of CMN for connected plants were mainly performed with few species of plants growing in pairs in microcosms and under controlled environmental conditions. Even under such controlled situation, the outcomes may still vary significantly, once benefits of connected plants may change according to host's physiological status, plants and fungal species involved, environment conditions, nutrient availability, etc. (Wagg et al., 2011). With this in mind, it is necessary to assess the most recent findings in literature and define still open questions, in order to guide upcoming studies in the area aimed to have a better understanding of possible plant-to-plant interactions via CMN and its effect shaping plants community. The present paper therefore evaluates results and theories of the functionality of CMN for plant-to-plant communication, especially, for resource exchange. Here, we also point the gaps of such studies in order to highlight especial points that need to be address in further studies.

### Source Sink Theory

In the source-sink model, the source is defined as the entity that can produce more of a given resource than it uses and the sink as the entity that has the potential/necessity to use more of a given resource than it produces (Heaton et al., 2012). The primary importance of plant–sink strength in governing the magnitude and direction of resource transfer through CMNs is illustrated in studies showing transfer of C to rapidly growing young EM trees with high transpiration rates, or to shaded seedlings with high respiration demands, increasing its survival and growth (Lekberg et al., 2010; Philip et al., 2010). Similarly, transfer of other resources, such as nitrogen (N), were also reported following a source-sink pattern (Montesinos-Navarro et al., 2017; Muneer et al., 2020). This mechanism has been proposed to increase the regenerative capacity of forest ecosystems (Teste et al., 2009, 2010). However, there are also reports of reduced transfer of C within a CMN to sink (shaded, defoliated, seedling) plants (Kytöviita et al., 2003; Walder et al., 2012), and even C transfer from sink (shaded) plants to source plants (Deslippe and Simard, 2011). Thus, a better understanding of the forces driving such interactions is required, since it has profound implications for our understanding of plant communities and competition. Depending on the species involved in the CMN and the possible effects for its fitness, it will drive forest community composition and dynamics (Beiler et al., 2010; Simard et al., 2015).

### Biological Market Theory

Asymmetry on resource allocation has been also demonstrated to increase competition between connected species (Merrild et al., 2013; Weremijewicz et al., 2016). Merrild et al. (2013) found that the growth suppression of small neighboring plants was diminished by clipping the shoots of large plants, which also increased the P uptake by interconnected small neighbors 6.5-fold. In order to exclude that suppression was caused by a general negative growth response, treatments including solitary vs. networked seedling was performed. In the referred study, suppression occurs only when seedlings were linked to the extraradical mycelium (ERM) of the large plant. Therefore, the authors concluded that the observed effects could solely be attributable to the CMN effect. However, such results has to be interpreted carefully, since inherent characteristics of plant species involved, such as growth rate, size, and root:shoot ratio, are likely to influence observed nutrient uptake.

Nevertheless, based on the observed results, an alternative theory has been proposed to elucidate such effects, the biological market theory. This theory is based on the assumption that both, plant and fungi, are able to detect variation in quality and amount of the resource supplied by their partner, allowing them to adjust their own resource allocation according to its gains (Kiers et al., 2011; Walder and van der Heijden, 2015; Werner and Kiers, 2015; Wang et al., 2019). Kiers et al. (2011) used molecular markers and stable isotope probing to track C flow from *Medicago truncatula* hosts into fungal RNA of roots colonized by mixed AM fungal communities with different cooperative behavior to the host plant. The authors found greater C enrichment in the most beneficial fungal species, suggesting a preferential allocation of C by the host, operating in a small spatial scale. The opposite flux was also observed, in which the fungi delivered more P for the host, which provided more C to fungi. Fellbaum et al. (2014) also evidenced fungal discrimination by greater N allocation to the host under elevated C allocation. If “rewards” indeed are reciprocal between mycorrhizal fungi and host plants, larger plants are supposed to obtain larger amounts of limiting nutrients by the fungal networks once they can produce and allocate much more C to the fungal partner. Increasing competition and suppressing growth of smaller individuals thus makes CMN a stronghold to avoid outcompeting its own kind.

It is important to note that the market theory proposed by some authors goes in an opposite direction to what was stated in the “source-sink” theory presented above. Neither theory should be defined as an universal framework to explain resource exchange in the mycorrhizal association nor predict plant interactions within a CMN, since the outcome of such interactions may vary with environmental conditions, functional diversity, competition for surplus resources, reciprocity and sink strength. Therefore, the effect of each variable should be tested separately and considered into the proposed models in order to define a more universal framework.

## Underground Connectivity

Both the source-sink theory and the market theory relies on the prerequisite of an underground connectivity of plants via CMN. In general, ecologists agree on the definition of CMN as a physical linkage among plants via the mycelia of the mycorrhiza fungi and that this linkage is common in nature (Simard and Durall, 2004; Simard et al., 2012; Hoeksema, 2015). However, this premise comes from observations that species of AM fungi are often compatible with multiple host plant species. In addition, Giovannetti et al. (2001) have demonstrated the ability of genetically compatible hyphae to anastomose (fusion), with disappearance of hyphal walls and exchange of cytoplasm and nuclei (Barreto de Novais et al., 2017). Both findings suggesting that CMNs are probably ubiquitous, although confirmation of such assumption still requires direct evidence for these linkages in the field. In this context, plants of same and different species have been reported sharing same fungi species or even same genet in several ecosystems (Simard et al., 2012; Beiler et al., 2015). Some authors have estimated the potential of plants to become interconnected by evaluating the similarity between mycorrhizal community composition, assuming a greater similarity when plants are connected through a CMN (Beiler et al., 2010; Diédhiou et al., 2010). (Beiler et al., 2010), for example, evaluated the distribution of genets of two species of ECM fungi (*Rhizopogon vesiculosus* and *R. vinicolor*) among roots of individual trees of Interior Douglas-fir (*Pseudotsuga menziesii*) as a network link. The authors proposed a model where trees of different ages were connected in a scale-free architecture and the larger trees served as hubs of nutrition, favoring understory regeneration, and functional continuity in the stand.

These achievements were of great importance to demonstrate the complexity of the CMN and the number and diversity of individuals that are potentially linked, resulting in a multitude of interactions involving multiple generations. However, sharing compatible species or even the same genets, does not necessarily indicate a direct connection among the host plants. Collembolas, for example, are known to feed on fungal hyphae. Such as AM fungi, they are widespread and abundant in the soil (Ekblad et al., 2013; Ngosong et al., 2014). By grazing the hyphae of a genet connecting two or more plants, this genet can still be identified in the roots of those plants although they would no longer be connected (Rotheray et al., 2008; Beiler et al., 2010). This is one of the examples of CMN disruption that could occur in the soil, and would be hard to identify (Wu et al., 2005; Beiler et al., 2015). Consequently, technical difficulties in proving hyphal connections between plants are the main obstacle when identifying whether any observed effect is really an intrinsic property of a CMN.

Therefore, it is also important to prove the extent and continuity of the mycelial network, together with mechanisms driving such connections and its consequences for plant fitness. In this context, there are few non-destructive methods for mycelium network observation, especially for AM fungi, mostly by the use of root observation chambers (Mikkelsen et al., 2008; Gyuricza et al., 2010) and *in vitro* dual systems (Kiers et al., 2011; Van't Padje et al., 2021). Such studies have nicely demonstrated the architecture of the extraradical mycelium of the fungi connecting two neighboring plants, but yet the relative importance of such network under realistic conditions is frequently under debate. For experiments developed in the forest, many interferences are found and the effects and mechanisms involved in the CMN cannot be excluded from other effects, such as positive and negative plant-soil feedback due to modulation of soil microbiota and biogeochemical cycles or even by production of roots exudates that might affect growth of nearby plants (Hu et al., 2018). Therefore, mycorrhizae studies still face challenges, raising questions if the data represents a natural situation, since there are no guarantees that evaluated effects are caused by mycorrhizae network.

## Mechanisms Involved for Plant Interaction VIA CMN

Currently, the mechanisms that drive benefits and competitive interactions between plants involved into a CMN has been under debate (e.g., Fellbaum et al., 2012; Bücking et al., 2016), raising diverse theories about the mechanism in these associations. The first one is based on the assumption that established mycorrhizal plants would facilitate mycorrhization of neighboring seedlings, acting as an inoculum and C source. In this case, seedlings would be able to join a CMN, which were already stablished and supported, in terms of translocation of reduced C by the older plants. Thus, seedling would be able to get access to limiting nutrients provided by the fungi without contributing with C supply to maintain the network. The second mechanism is based on the idea that CMN will act as conduits for interplant nutrient transfer (Gilbert and Johnson, 2017; Wipf et al., 2019). In this context, depending on how resources are distributed between connected plants, plants may either benefit by a more equilibrate distribution of resources or by increasing discrepancies of resources. In the first case, plants with higher nutritional conditions may donate excess of their resources to the receiver plants by a direct transfer. In the second case, resources might be distributed unequally favoring a certain group of individuals increasing therefore competitive interactions.

### Inoculum Source and Carbon Provision

Firstly, CMN may provide an inoculum source. Association with hyphae from the CMN can be much faster in comparison to soil spore bank, by the provision of an already established fungal inoculum source by the mature tree, permitting seedlings to quickly tap into a large soil resource pool that they could not access by their own (Bingham and Simard, 2012). Thus, this faster access to mycorrhizal services in the early plant stage, where mortality is high due to drought and biotic interactions, may be of critical importance, especially under harsh environmental conditions (Simard et al., 2012; Teste et al., 2015). In the experiment developed by Varga and Kytöviita (2016), the proportion of colonized seedlings by three different AM fungi was strongly related to the fungal species as well as to the source of inoculum. Seedlings inoculate much faster from nearby mycorrhized plants than from spores, despite a high spore density. This premise is also supported by some field experiments showing a positive relationship between the survival rate of seedling and its distance from the mature tree (McGuire, 2007; Grove et al., 2019). In addition, experiments involving barriers (e.g., mesh bags) or soil disturbance to manipulated seedling contact with CMN have shown higher seedling mortality when seedling are impeded to join the network (Nara, 2006; Pec et al., 2020).

Secondly, seedling may benefit from sharing a CMN with adult established tree since adult trees might provide much more C to sustain the network while seedling invest very little C and still obtain nutrients provided by the fungi. The maintenance of fungal symbiosis can be costly, resulting in a high C demand by the fungi for its development and activity (Smith and Read, 2010; Keymer et al., 2017; Rezáčová et al., 2017). In this context, sugars and lipids are the main C source derived from host plants transported to the fungal symbiont. Those C derived components will provide the fungi with the energy necessary for nutrient acquisition and the C skeleton for mycorrhizal growth (Bravo et al., 2017; Bezrutczyk et al., 2018). A benefit for seedlings would arise if larger trees pay the C cost required for the growth and maintenance of the CMN, so seedlings could potentially become mycorrhized and receive the benefits of this association without expending their own C for this (Diédhiou et al., 2010; Walder et al., 2012; Weremijewicz et al., 2016). In the study made by Högberg et al. (1999), for example, EM fungi connecting overstory pine trees with understory plants of different ages received 87–100% of their C from overstory trees and very little from understory trees. Walder et al. (2012) have shown a similar asymmetric pattern by using ^13^C of natural abundances between C_3_ and C_4_ plants without disturbing the system. The authors found that the C_4_ plant, which had the higher biomass, was invested more C to both fungal partner than the C_3_ plant but did not have a higher nutritional benefit. In this context, nutritional benefit strongly depended on the fungus involved in the CMN, in which *Rhizophagus irregularis* allocated nutrients preferentially to the C_3_ host plant while the CMN formed by *Glomus mosseae* were more balanced with respect to the nutrient allocation to both, C_3_ and C_4_, host plants. This demonstrate that C investment and nutritional benefit are not necessarily tightly linked and that some plant species can receive disproportional benefits from CMN. It is important to note that these experiments indicate that disproportional C investment by one plant does not necessarily mean a disadvantage for the other plant, especially when the cost of C is negligible for the main C donor.

### Mycorrhiza Network as Conduits for Interplant Resources Transfer

The premise of a possible nutrient transfer through a physical connection established by CMN may be of great importance in agricultural, where redistribution of symbiotic costs and benefits between individuals of the same or different plant species could increase growth of connected plants and therefore reduce amounts of chemical fertilizer input (Pena et al., 2013; Jansa et al., 2019). However, if a direct transfer of photoassimilates and nutrients between plants occurs via CMN is particularly controversially discussed (Bever et al., 2010; Courty et al., 2010). Such transfers have been frequently reported in field and laboratory experiments using labeling compounds to trace the fate of nutrients in plants connect by a CMN, trying to demonstrate belowground resource transfer between plants of same and different species is facilitated by mycorrhizal fungi (Teste et al., 2009; Deslippe and Simard, 2011; He et al., 2019; Fernandez et al., 2020).

In earlier studies, this mechanism was mainly observed in mycoheterotrophic plants, which are partly or entirely non-photosynthetic and indirectly parasitize green plants via CMN. These non-photosynthetic plants, also called epiparasites, associate with AM fungi emanating from the roots of surrounding green plants, therefore having access to C provided by those plants, together with other resources (Bidartondo et al., 2002; Girlanda et al., 2006; Selosse and Roy, 2009). In addition to mycoheterotrophic plants, some green orchids or small green perennial shrubs from the Ericaceae family have also been shown to receive considerable amounts of C from their mycorrhizal fungi (Selosse and Roy, 2009; Selosse et al., 2016). Those studies have raised the attention for the existence of a network where unrelated plants are able to transfer elemental compounds via shared fungal symbionts.

The mycorrhizal fungi which associates with mycoheterotrophic plants and green orchids usually belong to a diverse fungal taxa that also form mycorrhizae association with phototrophic tree roots (Zimmer et al., 2008; Waterman et al., 2013; Brundrett and Tedersoo, 2018). Since C transfer were observed between mycoheterotrophic and green plants and the same fungi species connecting those plants can also colonize several phototrophic trees, theories were raised regarding the possible C allocation between phototrophic trees as well. If such networks could act as a direct pathway of C and nutrients between green plants, this could play an important role for plant to plant interactions (Selosse and Roy, 2009; Smith and Read, 2010). Once C is an important resource for fungi growth, C allocation between plants would go to an opposite direction of the natural C flux commonly accepted in the symbiosis, which is from plant to fungi. In this case, one of the host plants would provide fungi with C and the fungi would not incorporate but channel this C through a neighboring plant. Some researchers believe that it might happen when networking fungus can acquire more C than it is required for its own fitness, therefore it may supply the excess to other plants in need (Gorzelak et al., 2015; Prescott et al., 2020). This has been suggested as a mechanism from the fungi to ensure survival of its host plants and therefore its access to multiple C supply, in case of a potential loss of one of the hosts (Gorzelak et al., 2015; Bücking et al., 2016). Some authors raised this theorem by using experiments involving high and low quality plants connected into a CMN (Kiers et al., 2011; Fellbaum et al., 2014; Bücking et al., 2016). In this context, the quality of a host is determined by its C investment into the mycorrhiza, in which low quality hosts have a reduced investment while high quality host can produce and allocate higher amounts of C to fungi partner. In previous studies, shading have been frequently used to reduce the plant's ability to produce C compounds to be exchanged by limiting nutrients. In such experiments, although a discrimination between plants was observed leading to higher resources (such as N and P) allocation to high quality host of the network, the fungi also transferred nutrients for the low quality host and maintained a high colonization rate in these plants (Kiers et al., 2011; Fellbaum et al., 2014; Bücking et al., 2016). Those mechanisms shows a possible strategy from the fungi in maintaining both high and low quality host into the network, to ensure that the possible loss of a high quality host is not harmful for its survival. This might be an important mechanism for fungi survival, especially under variable environments, as suggested first by Perry et al. (1989) and Wilkinson (1998).

In this context, Simard et al. (1997a) was one of the first to demonstrate a bi-directional flux of C between two autotrophic plants, Douglas-fir (*P. menziesii*) and paper birch (*Betula papyrifera*) species, sharing an EM network. Here, a great amount of C was observed to be exchanged between the plant species, with no net gain for any one of them in the end. However, in the second year of study, Simard et al. (1997b) observed a net gain of C by one of the species independently of full, partial or deep shade light intensity. However, some methodological issues regarding the experimental design of this study was unraveled later by Robinson and Fitter (1999), raising doubts regarding the ecological relevance of CMN-facilitated resource transfer. Simard et al. (1997b) used a double labeling technique (^14^C and ^13^C) to track C exchange between plants connected by an EM network in the field and calculate proportions of C received by each individual. However, not only EM connected plants received the applied C, but AM surrounding plants not connected to the network had access to labeled C too. That demonstrate that the movement of C between plants were not necessarily exclusively by mycorrhizal links, but could have reached neighboring plant by different pathways. This is especially likely to occur when no physical barriers are used in the experiments.

Robinson and Fitter (1999) also suggested that C transferred from neighboring photosynthetic active plant to hyphae within the roots of C-stressed plants is probably a strategy of the fungi for its own growth and survival, with minor consequences for plant communities. Teste et al. (2010) using a different experimental design also showed a low net C transfer between Douglas-fir seedlings in the field relative to total C uptake by photosynthesis. The significance of the amounts transferred have been repeatedly questioned in other works (Teste et al., 2009; Philip et al., 2010; Pickles et al., 2017), raising a center debate on whether the extent of net transfer from one plant to another is sufficiently large to affect significantly plant fitness and predict communities' dynamics. In addition, there are also reports about the accumulation of C partially or entirely in mycorrhizal roots of receiver plants, probably in fungal tissues, and not detected on shoots even under situations where root to shoot C flow is encouraged by clipping or shadding (Robinson and Fitter, 1999; Pfeffer et al., 2004; Lekberg et al., 2010). However, some authors argue that the movement of C to receiver plant, even without transfer into plant tissues, is still an important subsidy to meet the nutrient requirements of the plant, especially under stress conditions (Bever et al., 2010; Teste et al., 2015).

Mycorrhizal networks have also been frequently reported to play an important role for belowground transfer of N among plants, but as for C different studies lead to contradicting results. Patterns of N transfer have been studied using natural abundance (δ^15^N) or ^15^N-enriched techniques. For the ^15^N-enriched techniques, fertilizer is applied directly to the growth media of the N donor root or directly to the N donor plant by exposure to ^15^N_2_ (in case of experiments using N-fixing bacteria as an additional symbiont to host plant) or foliar spray or petiole injection of labeled ^15^N (${\text{NH}}_{4}^{+}$, ${\text{NO}}_{3}^{-}$, or urea). In early studies of several intercropping systems, a substantial one-way N transfer was demonstrated via a source-sink gradient from N_2_ fixing plants to non-N_2_ fixing plants, within a range of 20–50% (He et al., 2009). However, when a bi-directional flux was considered it was possible to note a greater flux of N from non-fixing plants to N-fixing plants, contradicting the source-sink theory initially proposed by this system (He et al., 2005, 2009; Pirhofer-Walzl et al., 2012).

Moreover, a transfer between N_2_ non-fixing donors and receiver plants of varying amount of N has also been observed. The transfer of N usually was reported to be lower than 5 % of N added by pulse labeling, while the direction of transport was largely found to be correlated with plant size (Teste et al., 2009, 2015; He et al., 2019) or plant physiology (Meding and Zasoski, 2008; Weremijewicz et al., 2018). Teste et al. (2009) also suggested that C and N move together in form of amino acids, once the stoichiometry of the relative amounts of C and N transferred was similar of this compound, but they were never identified (Simard et al., 2015).

**Figure 1:**
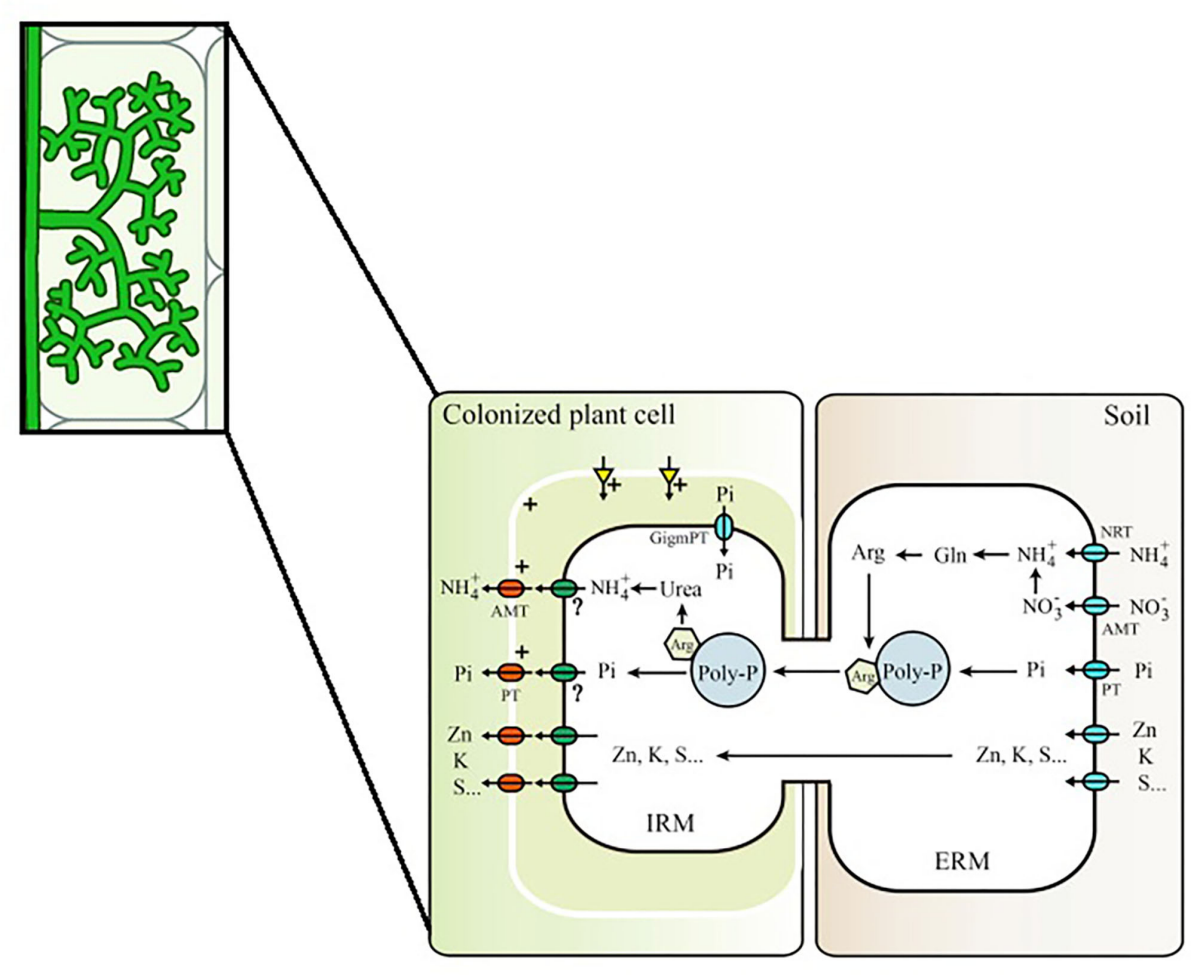
Mycorrhizal pathways for Pi and N in AM Symbiosis. In the mycorrhizal pathway, Pi is assimilated directly via phosphate importers while ammonium (${\text{NH}}_{4}^{+}$) and/or nitrate (${\text{NO}}_{3}^{-}$) are assimilated into glutamine (Gln) and then into arginine (Arg). Assimilation will generate excess H^+^ or OH^−^ with ${\text{NH}}_{4}^{+}$ and ${\text{NO}}_{3}^{-}$, respectively. Phosphate is mainly transported in the form of polyphosphate (Poly-P) granules, which is negatively charged, making possible it association with arginine and metal ions for further transportation to the IRM. Phosphate (Pi) and ${\text{NH}}_{4}^{+}$ transporters from the intraradical mycelium (IRM) to the interfacial apoplast are still unknown and therefore marked with a “?,” requiring further study (modified from Wang et al., 2017).

Interestingly, the idea of plant-to-plant transfer implies that N may flow in the “opposite” direction of what is widely known to occur. In the context of nutrient uptake, the current model suggests that P and N acquired from surrounding soil by the ERM of the fungi are transferred to the intraradical mycelium (IRM) as polyphosphate (polyP) and arginine, respectively, stored later on in vacuoles (Hijikata et al., 2010; Bücking and Kafle, 2015). Once in the IRM, polyp, and arginine are catabolized and Pi and ammonium are released and transported to the plants through transporters present in the periarbuscular membrane (Breuillin-Sessoms et al., 2015; Wang et al., 2017; Figure 1). Therefore, for plant-to-plant transfer, N should be transferred in the opposite direction: from plant to the IRM via transporters in the periarbuscular membrane, from IRM transferred to the ERM of the fungi and then again to the IRM of the receiver plant to be assimilated. Although many studies have been made in order to prove such transfer via connected hyphae (please check Supplementary Table 1 for some of those studies), such fluxes were never described anywhere. In the studies presented in Supplementary Table 1, it is also possible to observe that amount of N transferred via CMN is quite variable, probably due to differences in the experimental design and the choice of plant and fungi combination. In addition, transfer exclusively via mycelium connection in comparison to other possible are not distinguishable, especially in those studies in which a mesh barrier is not used to prevent roots intermingle and flow of soil solution.

### Direct Vs. Indirect Transfer

Mechanistically, AM fungi can facilitate the transfer of N between plants by creating direct mycelial connections between donors and receivers (Høgh-Jensen, 2006; Meng et al., 2015; He et al., 2019). When it comes to resource allocation through CMN, it is easy to notice a disagreement regarding its concept within published papers, even most recent ones. On the one hand some authors report a transport of nutrients via CMN exclusively via connected hyphae, thus describing hyphae as “pipelines” for resources (Klein et al., 2016; Van Der Heijden, 2016). On the other hand, other authors describe nutrient transfer to occur at least additionally via an indirect pathway. In such pathway, compounds are exuded or leaked into the soil pool by the roots or associated hyphae of one plant and then picked up by the roots or associated hyphae of a neighboring plant or even by other microorganisms present in the soil (Jansa et al., 2019; Fernandez et al., 2020; Fang et al., 2021). In this context, it is frequently stated in studies that CMN simply facilitate transfers between plants without further specification of the mode of transport, although this transport may occur by several pathways simultaneously between a single pair of plants (Wang et al., 2016; Fang et al., 2021).

For these indirect pathways, resources are vulnerable for potential disruptions, such as adsorption of nutrients to soil particles, immobilization and mineralization by surrounding microorganisms, biochemical transformation, and others (Philip et al., 2010; Simard et al., 2012). Thus, a direct pathway genuinely utilizing mycorrhizal hyphae would represent a potential conduit of resource sharing, in which resources would be free of disruption by leakage and re-assimilation by other microorganisms.

**Figure 2:**
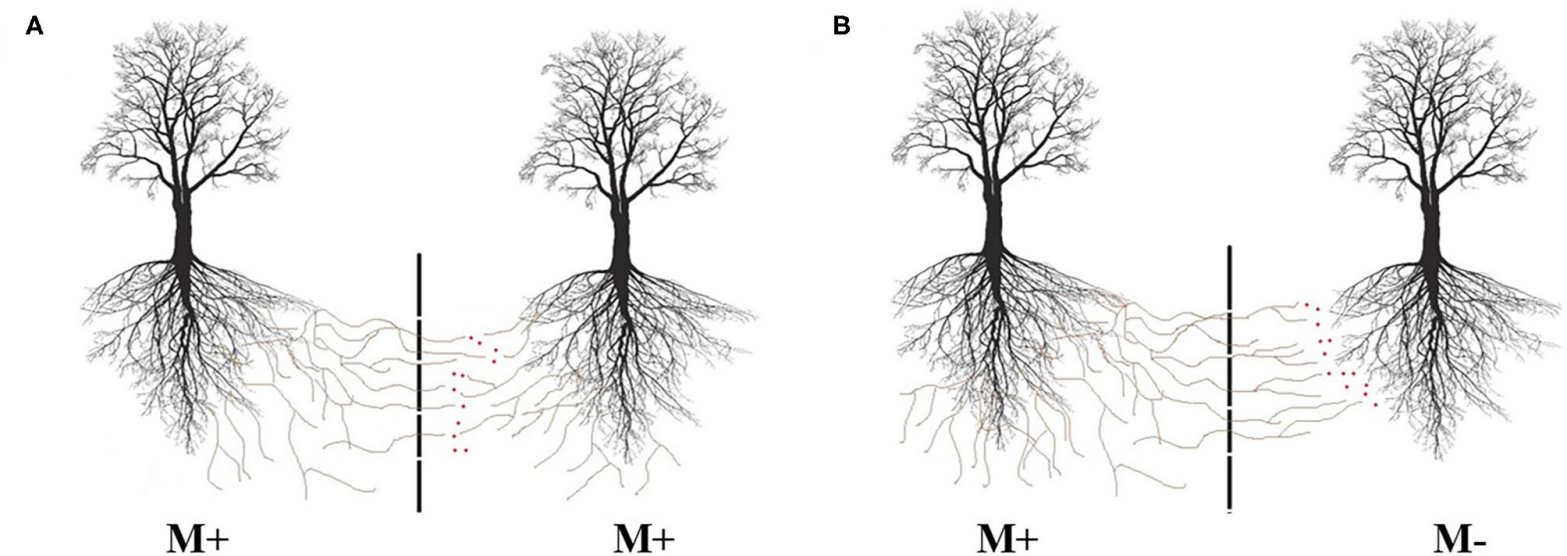
Possible movement of resources between networked plants, even when mesh barriers are used to avoid roots intermingle and flow of soil solution between connected plants. **(A)** Represents disrupted hyphal connections between two mycorrhizal (M+) plants and **(B)** possible transfer from a mycorrhized (M+) plant to a non-mycorrhized (M−) neighbor plant.

In field and laboratory studies, split root designs and root restrictive screening techniques have been used to determine the different pathways in interplant transfers (Xiao et al., 2004; He et al., 2005; Meng et al., 2015; Muneer et al., 2020). These designs can effectively prevent contact between individual host plant root systems, but they do not entirely prevent bulk flow or diffusive chemical movement in the soil water. Therefore, some experimental designs rely on air gaps to avoid diffusion over the soil solution flow while allowing the ingrowth of hyphae but not roots. This assures that all labeled compounds found in the receiver plant using in this system can be attributed to the mycorrhizal transport (Zhang et al., 2020; Andrino et al., 2021; Fang et al., 2021). However, these measures still do not exclude a transfer over indirect pathways, once transported resources by fungi mycelium can be released on neighboring plant compartment, leading the receiver plant to have access to resources without being connected (Figure 2). Moreover, connections among plants can hardly be directly visualized in soils of traditional pot experiments or even under field conditions.

Therefore, due to the technical difficulties to distinguish between transport pathways, it still remains unknown whether transfer occurs preferentially via direct hyphae connections or through indirect pathways. Creation of new experiment set-ups using new technologies to improve previous experiments should be developed for a more accurate idea about resource allocation and plant physiological responses that are truly accountable to CMN. Manipulation of the genes involved in setting up symbiotic associations between plant and fungi partner may help to differentiate the fungal effect in such networks (Merrild et al., 2013; Song et al., 2014). Mutant lines where the development of arbuscules is impaired and not functional are a promising starting point, and at least for *M. truncatula* such a mutant line is already known. Arbuscules are recognized as the main site of exchange, and comparing networks formed by wild type and mutant lines might lead to e better understanding of the effects of arbuscular network on the development of donor and receiver. Unfortunately, to our knowledge there are no such impaired mutant lines for EM fungi, therefore such studies are only possible for AM networks. In addition, some plant genera such Acacia, Alnus, Eucalyptus, Fraxinus, Populus, Salix, Shorea, and Uapaca are recognized to associate with both AM and EM fungi simultaneously (Teste et al., 2020), although frequency of each fungi type might differ along plant life. Much less research have been made in dual-mycorrhizae plants, and how AM and EM networks may affect connected plants differently. Altogether, such experiments could be helpful in order to achieve deeper understanding of mechanisms and processes behind CMN and its impact on plant community.

Nevertheless, it is important to keep in mind that, even if resources exchanges between plants takes place mainly via indirect pathways, receiver plants can still be favored by a facilitation of its access to resource coming from neighboring plants, which may anyway play a role in plant-to-plant interaction (Høgh-Jensen, 2006; Alaux et al., 2021).

### Role of Transfer for Plant Fitness

The simple movement of elements from one plant to another does not by itself indicate a net transfer able to represent an ecological advantage on plant fitness (Kytöviita et al., 2003; Bücking et al., 2016). Quantifying the contribution of each pathway to plant fitness is likewise a matter of discussion in most studies on CMN. However, quantification of nutrient and C fluxes exclusive to the fungal hyphae is difficult. To the best of our knowledge, there are only few quantitative information on the magnitude of C fluxes between plants sharing a CMN. In general, C transfer through CMN is not frequently considered a significant pathway for mobile C transfer among plants, although some authors suggest that even small amounts may be of great importance for receiver plant survival and development (Wu et al., 2001; Deslippe and Simard, 2011; Klein et al., 2016). This can be especially true if the receiver plants are seedlings (Nara, 2006; Booth and Hoeksema, 2010; Burke et al., 2018; Liang et al., 2021). Reported amounts of C vary from 0 up to 10% in literature (Teste et al., 2010; Lin et al., 2020). Simard et al. (1997b) was the first attempting to quantify a bidirectional flux of C between plants connected via EM network, in order to evaluate its ecology significance. The authors concluded that there was no net transfer between the species. However, the study raised debate in the literature due to its difficulty in extrapolating the data from young seedlings to mature tree and the use of relevant controls (Robinson and Fitter, 1999; Simard et al., 2012; Tedersoo et al., 2020). In addition, Simard et al. (1997b) concluded that it was not possible to distinguish whether the translocation occurred through interconnecting hyphae, soil pathways, or even both simultaneously, and, hence, did not really demonstrate the contribution of the CMN for C transfer.

A more recent approach was developed by Klein et al. (2016), attempting to evaluate C transfer between trees in a mature forest. They continuously labeled five 40-m-tall Norway spruce trees (*Picea abies*) as part of a 5-year free-air CO_2_ enrichment experiment (FACE) with ^13^C-depleted CO_2_. Despite the low difference in the δ^13^C ratios of canopy twigs, stems, and fine roots between labeled and unlabeled control (max. 2.6‰), the isotopic signal of neighboring trees belonging to same or different taxa (*Fagus sylvatica, Pinus sylvestris*, and *Larix decidua*) were than measured to evaluate C allocation. The authors claimed to find evidences that reciprocal C transfer indeed occurred between trees, as δ^13^C of fine roots of neighboring plants followed the same signal from the donor *Picea*. Most of the label was found in the fine roots, which was concluded to prove the participation of the mycorrhizae in the transfer. It was estimated that C derived from transfer represents 4% of net primary productivity.

Another point usually under discussion regarding C transfer is whether transferred C is taken up by the receiver plant for its own growth or, contrastingly, whether the C is mainly kept in the roots, probably incorporated into fungal structures, therefore not representing a meaningful advantage for the receiver plant. This was evaluated, for example, by Waters and Borowicz (1994) and Fitter et al. (1998). They assumed that by clipping the aboveground parts of living plants, additional C would be required and translocated from the roots to the re-growing clipped shoots. However, in neither of the experiments labeled C was found in the re-growing shoots of the receiver plants. Thus, the authors concluded that the transferred C remained in fungal structures. The opposite was found by Song et al. (2015) who reported labeled ^13^C in the shoots of the receiver plant. Another difference in the mentioned studies is that, in the experiment developed by Song et al. (2015), C transfer from donor to receiver plant increased by increasing defoliation of donor plant. This has been suggested as an effect of the sink-source strength of the connected plants. The authors concluded authors that defoliation could have stimulated interior Douglas-fir donor to rapidly export labile C from enriched roots to the CMN, while the rapid growth rate of ponderosa pine would created a large sink. Nevertheless, even if it is assumed that mycorrhizae might be able to transfer C from one plant to its neighbors, it remains unclear if the amounts of the transferred elements are of any significance to the receiver plant. If this amount is viable for the receiver plant, a process understanding of the switch between fungal storage and delivery to the plant is still required.

Equally contradictory is the magnitude of N transfer reported in the literature. In grassland ecosystems, N transfer was reported to vary from 0 to 72% under field conditions, while it is less variable in agroforestry ecosystems, ranging from 0 up to 16%, depending of the conditions under which the experiments were performed (e.g., in pots, field, etc.; Marty et al., 2009; Chapagain and Riseman, 2014; Meng et al., 2015; Zhang et al., 2020). In general, the high variability in the literature may reflect many different factors that might interfere in plant-to-plant interaction, such as differences in environmental conditions, in the different experimental setups, or plant and fungi combinations, soil nutrient supply, additional stress conditions added (e.g., nutrients deficiency, drought, shading, etc.), and the general experimental design (e.g., field, pot or microcosmos experiments). In addition, like for C, quantification of N transfer via interconnecting hyphae is not distinguishable from other pathways (Montesinos-Navarro et al., 2016; Fang et al., 2021). The distinction and relative importance of the different pathways determines the strength, direction, and outcome of interactions among plants and soil organisms, requiring new technologies and ideas to address such issues. Nevertheless, the many researches made on this topic so far developed different hypotheses that could give us some hints on how CMN would affect plant-plant interaction.

## Conclusions

Despite of the great progress in understanding the effect of mycorrhiza network for plant-to-plant interactions, and how this might affect mycorrhizal communities, there are still important questions to be answered in future researches. Resource allocation between connected plants thereby drew the largest attention of the scientific community. Many possible effects of such transfers of resources have been described, but contrasting results were frequently found. Labeled experiments using C and N isotopes have revealed that under certain conditions a movement of such resources between donor and receiver plants seem to happen, but none of them could demonstrate unequivocally that the transfer occurred preferentially through the direct mycorrhizal pathway and not over the soil solution or simply over exudates. Moreover, quantification of this transfer demonstrated to be an even bigger challenge. Therefore, the real effect of the CMN in shaping plant communities is still not clear. Further research involving new experiment set-ups and new technologies to improve previous experiments should be developed for a more accurate idea about resource allocation and plant physiological responses that are truly accountable to CMN. The use of mutant lines with manipulation of the genes involved in setting up symbiotic associations between plant and fungi partner together with labeling techniques to track resources translocation between connected plants can be used to differentiate the fungal effect in such networks. Effects exclusively to CMN for plant interactions may help us to understand plant community and ecosystem functioning.

## Author Contributions

AF, JB, and GG conceived the idea about the topic reviewed in this manuscript. AF wrote the manuscript. JB and GG contributed with corrections and comments and approved the submitted version. All authors contributed to the article and approved the submitted version.

## Funding

We want to thank the German Research Foundation (Deutsche Forschungsgemeinschaft) for the funding of this project in the framework of the DFG-GRK 1798 Signaling at the Plant-Soil Interface. The publication of this article was funded by the Open Access fund of Leibniz Universität Hannover.

## Conflict of Interest

The authors declare that the research was conducted in the absence of any commercial or financial relationships that could be construed as a potential conflict of interest.

## Publisher's Note

All claims expressed in this article are solely those of the authors and do not necessarily represent those of their affiliated organizations, or those of the publisher, the editors and the reviewers. Any product that may be evaluated in this article, or claim that may be made by its manufacturer, is not guaranteed or endorsed by the publisher.

## References

[B1] Alaux P. L. Zhang Y. Gilbert L. Johnson D. (2021). Can common mycorrhizal fungal networks be managed to enhance ecosystem functionality? Plants People Planet. 1–12. 10.1002/ppp3.10178

[B2] Andrino A. Guggenberger G. Sauheitl L. Burkart S. Boy J. (2021). Carbon investment into mobilization of mineral and organic phosphorus by arbuscular mycorrhiza. Biol. Fertil. Soils 57, 47–64. 10.1007/s00374-020-01505-5

[B3] Babikova Z. Gilbert L. Bruce T. J. Birkett M. Caulfield J. C. Woodcock C.. (2013). Underground signals carried through common mycelial networks warn neighbouring plants of aphid attack. Ecol. Lett. 16, 835–843. 10.1111/ele.12115 23656527

[B4] Barreto de Novais C. Pepe A. Siqueira J. O. Giovannetti M. Sbrana C. (2017). Compatibility and incompatibility in hyphal anastomosis of arbuscular mycorrhizal fungi. Sci. Agric. 74, 411–416. 10.1590/1678-992x-2016-024326630971

[B5] Beiler K. J. Durall D. M. Simard S. W. Maxwell S. A. Kretzer A. M. (2010). Architecture of the wood-wide web: *Rhizopogon* spp. genets link multiple Douglas-fir cohorts. New Phytol. 185, 543–553. 10.1111/j.1469-8137.2009.03069.x 19878460

[B6] Beiler K. J. Simard S. W. Durall D. M. (2015). Topology of tree-mycorrhizal fungus interaction networks in xeric and mesic Douglas-fir forests. J. Ecol. 103, 616–628. 10.1111/1365-2745.12387

[B7] Bever J. D. Dickie I. A. Facelli E. Facelli J. M. Klironomos J. Moora M.. (2010). Rooting theories of plant community ecology in microbial interactions. Trends Ecol. Evol. 25, 468–478. 10.1016/j.tree.2010.05.004 20557974 PMC2921684

[B8] Bezrutczyk M. Yang J. Eom J. S. Prior M. Sosso D. Hartwig T.. (2018). Sugar flux and signaling in plant-microbe interactions. Plant J. 93, 675–685. 10.1111/tpj.13775 29160592

[B9] Bidartondo M. I. Redecker D. Hijri I. Wiemken A. Bruns T. D. Domínguez L.. (2002). Epiparasitic plants specialized on arbuscular mycorrhizal fungi. Nature 419, 389–392. 10.1038/nature01054 12353033

[B10] Bingham M. A. Simard S. (2012). Ectomycorrhizal networks of *Pseudotsuga menziesii* var. glauca trees facilitate establishment of conspecific seedlings under drought. Ecosystems 15, 188–199. 10.1007/s10021-011-9502-2

[B11] Bingham M. A. Simard S. W. (2011). Do mycorrhizal network benefits to survival and growth of interior Douglas-fir seedlings increase with soil moisture stress? Ecol. Evol. 1, 306–316. 10.1002/ece3.24 22393502 PMC3287316

[B12] Booth M. G. Hoeksema J. D. (2010). Mycorrhizal networks counteract competitive effects of canopy trees on seedling survival. Ecology 91, 2294–2302. 10.1890/09-1139.1 20836451

[B13] Bravo A. Brands M. Wewer V. Dörmann P. Harrison M. J. (2017). Arbuscular mycorrhiza-specific enzymes FatM and RAM 2 fine-tune lipid biosynthesis to promote development of arbuscular mycorrhiza. New Phytol. 214, 1631–1645. 10.1111/nph.14533 28380681

[B14] Breuillin-Sessoms F. Floss D. S. Gomez S. K. Pumplin N. Ding Y. Levesque-Tremblay V.. (2015). Suppression of arbuscule degeneration in *Medicago truncatula* phosphate transporter4 mutants is dependent on the ammonium transporter 2 family protein AMT2; 3. Plant Cell 27, 1352–1366. 10.1105/tpc.114.131144 25841038 PMC4558683

[B15] Brundrett M. C. (2009). Mycorrhizal associations and other means of nutrition of vascular plants: understanding the global diversity of host plants by resolving conflicting information and developing reliable means of diagnosis. Plant Soil 320, 37–77. 10.1007/s11104-008-9877-9

[B16] Brundrett M. C. Tedersoo L. (2018). Evolutionary history of mycorrhizal symbioses and global host plant diversity. New Phytol. 220, 1108–1115. 10.1111/nph.14976 29355963

[B17] Bücking H. Kafle A. (2015). Role of arbuscular mycorrhizal fungi in the nitrogen uptake of plants: current knowledge and research gaps. Agronomy 5, 587–612. 10.3390/agronomy5040587

[B18] Bücking H. Mensah J. A. Fellbaum C. R. (2016). Common mycorrhizal networks and their effect on the bargaining power of the fungal partner in the arbuscular mycorrhizal symbiosis. Commun. Integr. Biol. 9:e1107684. 10.1080/19420889.2015.1107684 27066184 PMC4802747

[B19] Burke D. J. Klenkar M. K. Medeiros J. S. (2018). Mycorrhizal network connections, water reduction, and neighboring plant species differentially impact seedling performance of two forest wildflowers. Int. J. Plant Sci. 179, 314–324. 10.1086/696686

[B20] Chapagain T. Riseman A. (2014). Barley-pea intercropping: effects on land productivity, carbon and nitrogen transformations. Field Crops Res. 166, 18–25. 10.1016/j.fcr.2014.06.014

[B21] Courty P. E. Buée M. Diedhiou A. G. Frey-Klett P. Le Tacon F. Rineau F.. (2010). The role of ectomycorrhizal communities in forest ecosystem processes: new perspectives and emerging concepts. Soil Biol. Biochem. 42, 679–698. 10.1016/j.soilbio.2009.12.006

[B22] Deslippe J. R. Simard S. W. (2011). Below-ground carbon transfer among *Betula nana* may increase with warming in Arctic tundra. New Phytol. 192, 689–698. 10.1111/j.1469-8137.2011.03835.x 21797881

[B23] Diédhiou A. G. Selosse M. A. Galiana A. Diabaté M. Dreyfus B. Bâ A. M.. (2010). Multi-host ectomycorrhizal fungi are predominant in a Guinean tropical rainforest and shared between canopy trees and seedlings. Environ. Microbiol. 12, 2219–2232. 10.1111/j.1462-2920.2010.02183.x 21966915

[B24] Ekblad A. Wallander H. Godbold D. L. Cruz C. Johnson D. Baldrian P.. (2013). The production and turnover of extramatrical mycelium of ectomycorrhizal fungi in forest soils: role in carbon cycling. Plant Soil 366, 1–27. 10.1007/s11104-013-1630-3

[B25] Fang L. He X. Zhang X. Yang Y. Liu R. Shi S.. (2021). A small amount of nitrogen transfer from White Clover to Citrus seedling via common arbuscular mycorrhizal networks. Agronomy 11:32. 10.3390/agronomy11010032

[B26] Fellbaum C. R. Gachomo E. W. Beesetty Y. Choudhari S. Strahan G. D. Pfeffer P. E.. (2012). Carbon availability triggers fungal nitrogen uptake and transport in arbuscular mycorrhizal symbiosis. Proc. Nat. Acad. Sci. U.S.A. 109, 2666–2671. 10.1073/pnas.1118650109 22308426 PMC3289346

[B27] Fellbaum C. R. Mensah J. A. Cloos A. J. Strahan G. E. Pfeffer P. E. Kiers E. T.. (2014). Fungal nutrient allocation in common mycorrhizal networks is regulated by the carbon source strength of individual host plants. New Phytol. 203, 646–656. 10.1111/nph.12827 24787049

[B28] Fernandez M. Malagoli P. Vernay A. Ameglio T. Balandier P. (2020). Below-ground nitrogen transfer from oak seedlings facilitates Molinia growth: 15 N pulse-chase labelling. Plant Soil 423, 59–85. 10.1007/s11104-020-04473-9

[B29] Fitter A. H. Graves J. D. Watkins N. K. Robinson D. Scrimgeour C. (1998). Carbon transfer between plants and its control in networks of arbuscular mycorrhizas. Funct. Ecol. 12, 406–412. 10.1046/j.1365-2435.1998.00206.x21836016

[B30] Gilbert L. Johnson D. (2017). Plant-plant communication through common mycorrhizal networks. Adv. Bot. Res. 82, 83–97. 10.1016/bs.abr.2016.09.00120967206

[B31] Giovannetti M. Fortuna P. Citernesi A. S. Morini S. Nuti M. P. (2001). The occurrence of anastomosis formation and nuclear exchange in intact arbuscular mycorrhizal networks. New Phytol. 151, 717–724. 10.1046/j.0028-646x.2001.00216.x 33853252

[B32] Girlanda M. Selosse M. A. Cafasso D. Brilli F. Delfine S. Fabbian R.. (2006). Inefficient photosynthesis in the Mediterranean orchid *Limodorum abortivum* is mirrored by specific association to ectomycorrhizal Russulaceae. Mol. Ecol. 15, 491–504. 10.1111/j.1365-294X.2005.02770.x 16448415

[B33] Gorzelak M. A. Asay A. K. Pickles B. J. Simard S. W. (2015). Inter-plant communication through mycorrhizal networks mediates complex adaptive behaviour in plant communities. AoB Plants 7:plv050. 10.1093/aobpla/plv050 25979966 PMC4497361

[B34] Grove S. Saarman N. P. Gilbert G. S. Faircloth B. Haubensak K. A. Parker I. M. (2019). Ectomycorrhizas and tree seedling establishment are strongly influenced by forest edge proximity but not soil inoculum. Ecol. Appl. 29:e01867. 10.1002/eap.1867 30710404

[B35] Gyuricza V. Thiry Y. Wannijn J. Declerck S. Dupré de Boulois H. (2010). Radiocesium transfer between *Medicago truncatula* plants via a common mycorrhizal network. Environ. Microbiol. 12, 2180–2189. 10.1111/j.1462-2920.2009.02118.x 21966912

[B36] He X. Critchley C. Ng H. Bledsoe C. (2005). Nodulated N2-fixing *Casuarina cunninghamiana* is the sink for net N transfer from non-N2-fixing *Eucalyptus maculata* via an ectomycorrhizal fungus *Pisolithus* sp. using 15NH4+ or 15NO3– supplied as ammonium nitrate. New Phytol. 167, 897–912. 10.1111/j.1469-8137.2005.01437.x 16101925

[B37] He X. Xu M. Qiu G. Y. Zhou J. (2009). Use of 15N stable isotope to quantify nitrogen transfer between mycorrhizal plants. J. Plant Ecol. 2, 107–118. 10.1093/jpe/rtp015

[B38] He Y. Cornelissen J. H. Wang P. Dong M. Ou J. (2019). Nitrogen transfer from one plant to another depends on plant biomass production between conspecific and heterospecific species via a common arbuscular mycorrhizal network. Environ. Sci. Pollut. Res. 26, 8828–8837. 10.1007/s11356-019-04385-x 30712202

[B39] Heaton L. Obara B. Grau V. Jones N. Nakagaki T. Boddy L.. (2012). Analysis of fungal networks. Fungal Biol. Rev. 26, 12–29. 10.1016/j.fbr.2012.02.001

[B40] Hijikata N. Murase M. Tani C. Ohtomo R. Osaki M. Ezawa T. (2010). Polyphosphate has a central role in the rapid and massive accumulation of phosphorus in extraradical mycelium of an arbuscular mycorrhizal fungus. New Phytol. 186, 285–289. 10.1111/j.1469-8137.2009.03168.x 20409186

[B41] Hoeksema J. D. (2015). Experimentally testing effects of mycorrhizal networks on plant-plant interactions and distinguishing among mechanisms, in Mycorrhizal Networks (Dordrecht: Springer), 255–277. 10.1007/978-94-017-7395-9_9

[B42] Högberg P. Högberg M. N. Quist M. E. Ekblad A. L. F. Näsholm T. (1999). Nitrogen isotope fractionation during nitrogen uptake by ectomycorrhizal and non-mycorrhizal *Pinus sylvestris*. New Phytol. 142, 569–576. 10.1046/j.1469-8137.1999.00404.x

[B43] Høgh-Jensen H. (2006). The nitrogen transfer between plants: an important but difficult flux to quantify. Plant Soil 282, 1–5. 10.1007/s11104-005-2613-9

[B44] Hu L. Robert C. A. Cadot S. Zhang X. Ye M. Li B.. (2018). Root exudate metabolites drive plant-soil feedbacks on growth and defense by shaping the rhizosphere microbiota. Nat. Commun. 9, 1–13. 10.1038/s41467-018-05122-7 30013066 PMC6048113

[B45] Jansa J. Forczek S. T. Rozmoš M. Püschel D. Bukovská P. Hršelová H. (2019). Arbuscular mycorrhiza and soil organic nitrogen: network of players and interactions. Chem. Biol. Technol. Agric. 6, 1–10. 10.1186/s40538-019-0147-2

[B46] Kadowaki K. Yamamoto S. Sato H. Tanabe A. S. Hidaka A. Toju H. (2018). Mycorrhizal fungi mediate the direction and strength of plant-soil feedbacks differently between arbuscular mycorrhizal and ectomycorrhizal communities. Commun. Biol. 1, 1–11. 10.1038/s42003-018-0201-9 30480098 PMC6244237

[B47] Keymer A. Pimprikar P. Wewer V. Huber C. Brands M. Bucerius S. L.. (2017). Lipid transfer from plants to arbuscular mycorrhiza fungi. Elife 6:e29107. 10.7554/eLife.29107.051 28726631 PMC5559270

[B48] Kiers E. T. Duhamel M. Beesetty Y. Mensah J. A. Franken O. Verbruggen E.. (2011). Reciprocal rewards stabilize cooperation in the mycorrhizal symbiosis. Science 333, 880–882. 10.1126/science.1208473 21836016

[B49] Klein T. Siegwolf R. T. W. Körner C. (2016). Belowground carbon trade among tall trees in a temperate forest. Science 352, 342–344. 10.1126/science.aad6188 27081070

[B50] Kytöviita M. M. Vestberg M. Tuomi J. (2003). A test of mutual aid in common mycorrhizal networks: established vegetation negates benefit in seedlings. Ecology 84, 898–906. 10.1890/0012-9658(2003)084[0898:ATOMAI]2.0.CO;2

[B51] Lekberg Y. Hammer E. C. Olsson P. A. (2010). Plants as resource islands and storage units-adopting the mycocentric view of arbuscular mycorrhizal networks. FEMS Microbiol. Ecol. 74, 336–345. 10.1111/j.1574-6941.2010.00956.x 20722732

[B52] Liang M. Shi L. Burslem D. F. Johnson D. Fang M. Zhang X.. (2021). Soil fungal networks moderate density-dependent survival and growth of seedlings. New Phytol. 230, 1688–1689. 10.1111/nph.17237 33506513

[B53] Lin C. Wang Y. Liu M. Li Q. Xiao W. Song X. (2020). Effects of nitrogen deposition and phosphorus addition on arbuscular mycorrhizal fungi of Chinese fir (*Cunninghamia lanceolata*). Sci. Rep. 10, 1–8. 10.1038/s41598-020-69213-6 32704060 PMC7378246

[B54] Marty C. Pornon A. Escaravage N. Winterton P. Lamaze T. (2009). Complex interactions between a legume and two grasses in a subalpine meadow. Am. J. Bot. 96, 1814–1820. 10.3732/ajb.0800405 21622302

[B55] McGuire K. L. (2007). Common ectomycorrhizal networks may maintain monodominance in a tropical rain forest. Ecology 88, 567–574. 10.1890/05-1173 17503583

[B56] Meding S. M. Zasoski R. J. (2008). Hyphal-mediated transfer of nitrate, arsenic, cesium, rubidium, and strontium between arbuscular mycorrhizal forbs and grasses from a California oak woodland. Soil Biol. Biochem. 40, 126–134. 10.1016/j.soilbio.2007.07.019

[B57] Meng L. Zhang A. Wang F. Han X. Wang D. Li S. (2015). Arbuscular mycorrhizal fungi and rhizobium facilitate nitrogen uptake and transfer in soybean/maize intercropping system. Front. Plant Sci. 6:339. 10.3389/fpls.2015.00339 26029236 PMC4429567

[B58] Merrild M. P. Ambus P. Rosendahl S. Jakobsen I. (2013). Common arbuscular mycorrhizal networks amplify competition for phosphorus between seedlings and established plants. New Phytol. 200, 229–240. 10.1111/nph.12351 23738787

[B59] Mikkelsen B. L. Rosendahl S. Jakobsen I. (2008). Underground resource allocation between individual networks of mycorrhizal fungi. New Phytol. 180, 890–898. 10.1111/j.1469-8137.2008.02623.x 18801003

[B60] Montesinos-Navarro A. Verdú M. Querejeta J. I. Sortibrán L. Valiente-Banuet A. (2016). Soil fungi promote nitrogen transfer among plants involved in long-lasting facilitative interactions. Perspect. Plant Ecol. Evol. Syst. 18, 45–51. 10.1016/j.ppees.2016.01.004

[B61] Montesinos-Navarro A. Verdú M. Querejeta J. I. Valiente-Banuet A. (2017). Nurse plants transfer more nitrogen to distantly related species. Ecology 98, 1300–1310. 10.1002/ecy.1771 28188633

[B62] Muneer M. A. Wang P. Lin C. Ji B. (2020). Potential role of common mycorrhizal networks in improving plant growth and soil physicochemical properties under varying nitrogen levels in a grassland ecosystem. Glob. Ecol. Conserv. 24:e01352. 10.1016/j.gecco.2020.e01352

[B63] Nara K. (2006). Ectomycorrhizal networks and seedling establishment during early primary succession. New Phytol. 169, 169–178. 10.1111/j.1469-8137.2005.01545.x 16390428

[B64] Ngosong C. Gabriel E. Ruess L. (2014). Collembola grazing on arbuscular mycorrhiza fungi modulates nutrient allocation in plants. Pedobiologia 57, 171–179. 10.1016/j.pedobi.2014.03.002

[B65] Pec G. J. Simard S. W. Cahill J. F. Karst J. (2020). The effects of ectomycorrhizal fungal networks on seedling establishment are contingent on species and severity of overstorey mortality. Mycorrhiza 30, 173–183. 10.1007/s00572-020-00940-4 32088844

[B66] Pena R. Simon J. Rennenberg H. Polle A. (2013). Ectomycorrhiza affect architecture and nitrogen partitioning of beech (*Fagus sylvatica* L.) seedlings under shade and drought. Environ. Exp. Bot. 87, 207–217. 10.1016/j.envexpbot.2012.11.005

[B67] Perry D. A. Margolis H. Choquette C. Molina R. Trappe J. M. (1989). Ectomycorrhizal mediation of competition between coniferous tree species. New Phytol. 112, 501–511. 10.1111/j.1469-8137.1989.tb00344.x 29265433

[B68] Pfeffer P. E. Douds D. D. Jr Bücking H. Schwartz D. P. Shachar-Hill Y. (2004). The fungus does not transfer carbon to or between roots in an arbuscular mycorrhizal symbiosis. New Phytol. 163, 617–627. 10.1111/j.1469-8137.2004.01152.x 33873744

[B69] Philip L. Simard S. Jones M. (2010). Pathways for below-ground carbon transfer between paper birch and Douglas-fir seedlings. Plant Ecol. Divers. 3, 221–233. 10.1080/17550874.2010.502564

[B70] Pickles B. J. Wilhelm R. Asay A. K. Hahn A. S. Simard S. W. Mohn W. W. (2017). Transfer of 13C between paired Douglas-fir seedlings reveals plant kinship effects and uptake of exudates by ectomycorrhizas. New Phytol. 214, 400–411. 10.1111/nph.14325 27870059

[B71] Pirhofer-Walzl K. Rasmussen J. Høgh-Jensen H. Eriksen J. Søegaard K. Rasmussen J. (2012). Nitrogen transfer from forage legumes to nine neighbouring plants in a multi-species grassland. Plant Soil 350, 71–84. 10.1007/s11104-011-0882-z

[B72] Prescott C. E. Grayston S. J. Helmisaari H. S. Kaštovská E. Körner C. Lambers H.. (2020). Surplus carbon drives allocation and plant-soil interactions. Trends Ecol. Evol. 35, 1110–1118. 10.1016/j.tree.2020.08.007 32928565

[B73] Read D. J. (1991). Mycorrhizas in ecosystems. Experientia 47, 376–391. 10.1007/BF01972080

[B74] Rezáčová V. Konvalinková T. Jansa J. (2017). Carbon fluxes in mycorrhizal plants, in Mycorrhiza-eco-Physiology, Secondary Metabolites, Nanomaterials, ed Horton T. R. (Cham: Springer), 1–21. 10.1007/978-3-319-57849-1_1

[B75] Rhodes C. J. (2017). The whispering world of plants:'the wood wide web'. Sci. Prog. 100, 331–337. 10.3184/003685017X14968299580423 28779763 PMC10365201

[B76] Robinson D. Fitter A. (1999). The magnitude and control of carbon transfer between plants linked by a common mycorrhizal network. J. Exp. Bot. 50, 9–13. 10.1093/jxb/50.330.9

[B77] Rotheray T. D. Jones T. H. Fricker M. D. Boddy L. (2008). Grazing alters network architecture during interspecific mycelial interactions. Fungal Ecol. 1, 124–132. 10.1016/j.funeco.2008.12.001

[B78] Schüßler A. Walker C. (2011). Evolution of the 'plant-symbiotic' fungal phylum, glomeromycota, in The Mycota XIV - Evolution of Fungi and Fungal-like Organisms, eds Pöggeler S. Wöstemeyer J. (Berlin: Springer), 163–185. 10.1007/978-3-642-19974-5_7

[B79] Seiwa K. Negishi Y. Eto Y. Hishita M. Masaka K. Fukasawa Y.. (2020). Successful seedling establishment of arbuscular mycorrhizal-compared to ectomycorrhizal-associated hardwoods in arbuscular cedar plantations. For. Ecol. Manage. 468:118155. 10.1016/j.foreco.2020.118155

[B80] Selosse M. A. Bocayuva M. F. Kasuya M. C. M. Courty P. E. (2016). Mixotrophy in mycorrhizal plants: extracting carbon from mycorrhizal networks, in Molecular Mycorrhizal Symbiosis, ed Martin F. (Berlin: Springer Verlag), 451–471. 10.1002/9781118951446.ch25

[B81] Selosse M. A. Roy M. (2009). Green plants that feed on fungi: facts and questions about mixotrophy. Trends Plant Sci. 14, 64–70. 10.1016/j.tplants.2008.11.004 19162524

[B82] Simard S. Asay A. Beiler K. Bingham M. Deslippe J. He X.. (2015). Resource transfer between plants through ectomycorrhizal fungal networks, in Mycorrhizal Networks (Dordrecht: Springer), 133–176. 10.1007/978-94-017-7395-9_5

[B83] Simard S. W. Beiler K. J. Bingham M. A. Deslippe J. R. Philip L. J. Teste F. P. (2012). Mycorrhizal networks: mechanisms, ecology and modelling. Fungal Biol. Rev. 26, 39–60. 10.1016/j.fbr.2012.01.001

[B84] Simard S. W. Durall D. M. (2004). Mycorrhizal networks: a review of their extent, function, and importance. Can. J. Bot. 82, 1140–1165. 10.1139/b04-116

[B85] Simard S. W. Jones M. D. Durall D. M. Perry D. A. Myrold D. D. Molina R. (1997a). Reciprocal transfer of carbon isotopes between ectomycorrhizal *Betula papyrifera* and *Pseudotsuga menziesii*. New Phytol. 137, 529–542. 10.1046/j.1469-8137.1997.00834.x 33863069

[B86] Simard S. W. Perry D. A. Jones M. D. Myrold D. D. Durall D. M. Molina R. (1997b). Net transfer of carbon between ectomycorrhizal tree species in the field. Nature 388, 579–582. 10.1038/4155723912260

[B87] Smith S. E. Read D. J. (2010). Mycorrhizal Symbiosis. Oxford: Academic Press.

[B88] Song Y. Y. Simard S. W. Carroll A. Mohn W. W. Zeng R. S. (2015). Defoliation of interior Douglas-fir elicits carbon transfer and stress signalling to ponderosa pine neighbors through ectomycorrhizal networks. Sci. Rep. 5, 1–9. 10.1038/srep08495 25683155 PMC4329569

[B89] Song Y. Y. Ye M. Li C. He X. Zhu-Salzman K. Wang R. L.. (2014). Hijacking common mycorrhizal networks for herbivore-induced defence signal transfer between tomato plants. Sci. Rep. 4, 1–8. 10.1038/srep03915 24468912 PMC3904153

[B90] Song Y. Y. Zeng R. S. Xu J. F. Li J. Shen X. Yihdego W. G. (2010). Interplant communication of tomato plants through underground common mycorrhizal networks. PLoS ONE 5:e13324. 10.1371/journal.pone.0013324 20967206 PMC2954164

[B91] Tedersoo L. Bahram M. Zobel M. (2020). How mycorrhizal associations drive plant population and community biology. Science 367:6480. 10.1126/science.aba1223 32079744

[B92] Teste F. P. Jones M. D. Dickie I. A. (2020). Dual-mycorrhizal plants: their ecology and relevance. New Phytol. 225, 1835–1851. 10.1111/nph.16190 31514244

[B93] Teste F. P. Simard S. W. Durall D. M. Guy R. D. Berch S. M. (2010). Net carbon transfer between *Pseudotsuga menziesii* var. glauca seedlings in the field is influenced by soil disturbance. J. Ecol. 98, 429–439. 10.1111/j.1365-2745.2009.01624.x

[B94] Teste F. P. Simard S. W. Durall D. M. Guy R. D. Jones M. D. Schoonmaker A. L. (2009). Access to mycorrhizal networks and roots of trees: importance for seedling survival and resource transfer. Ecology 90, 2808–2822. 10.1890/08-1884.1 19886489

[B95] Teste F. P. Veneklaas E. J. Dixon K. W. Lambers H. (2015). Is nitrogen transfer among plants enhanced by contrasting nutrient-acquisition strategies? Plant Cell Environ. 38, 50–60. 10.1111/pce.12367 24811370

[B96] Toju H. Sato H. Yamamoto S. Kadowaki K. Tanabe A. S. Yazawa S.. (2013). How are plant and fungal communities linked to each other in belowground ecosystems? A massively parallel pyrosequencing analysis of the association specificity of root-associated fungi and their host plants. Ecol. Evol. 3, 3112–3124. 10.1002/ece3.706 24101998 PMC3790555

[B97] Van Der Heijden M. G. (2016). Underground networking. Science 352, 290–291. 10.1126/science.aaf4694 27081054

[B98] Van Der Heijden M. G. Horton T. R. (2009). Socialism in soil? The importance of mycorrhizal fungal networks for facilitation in natural ecosystems. J. Ecol. 97, 1139–1150. 10.1111/j.1365-2745.2009.01570.x

[B99] Van't Padje A. Galvez L. O. Klein M. Hink M. A. Postma M. Shimizu T.. (2021). Temporal tracking of quantum-dot apatite across *in vitro* mycorrhizal networks shows how host demand can influence fungal nutrient transfer strategies. ISME J. 15, 435–449. 10.1038/s41396-020-00786-w 32989245 PMC8027207

[B100] Varga S. Kytöviita M. M. (2016). Faster acquisition of symbiotic partner by common mycorrhizal networks in early plant life stage. Ecosphere 7:e01222. 10.1002/ecs2.1222

[B101] Wagg C. Jansa J. Stadler M. Schmid B. Van Der Heijden M. G. (2011). Mycorrhizal fungal identity and diversity relaxes plant-plant competition. Ecology 92, 1303–1313. 10.1890/10-1915.1 21797158

[B102] Wagg C. Veiga R. van der Heijden M. G. (2015). Facilitation and antagonism in mycorrhizal networks, in Mycorrhizal Networks eds Varma A. Prasad R. Tuteja N. (Dordrecht: Springer), 203–226. 10.1007/978-94-017-7395-9_7

[B103] Wahbi S. Maghraoui T. Hafidi M. Sanguin H. Oufdou K. Prin Y.. (2016). Enhanced transfer of biologically fixed N from faba bean to intercropped wheat through mycorrhizal symbiosis. Appl. Soil Ecol. 107, 91–98. 10.1016/j.apsoil.2016.05.008

[B104] Walder F. Niemann H. Natarajan M. Lehmann M. F. Boller T. Wiemken A. (2012). Mycorrhizal networks: common goods of plants shared under unequal terms of trade. Plant Physiol. 159, 789–797. 10.1104/pp.112.195727 22517410 PMC3375941

[B105] Walder F. van der Heijden M. G. (2015). Regulation of resource exchange in the arbuscular mycorrhizal symbiosis. Nat. Plants 1, 1–7. 10.1038/nplants.2015.159 27251530

[B106] Wang G. Sheng L. Zhao D. Sheng J. Wang X. Liao H. (2016). Allocation of nitrogen and carbon is regulated by nodulation and mycorrhizal networks in soybean/maize intercropping system. Front. Plant Sci. 7:1901. 10.3389/fpls.2016.01901 28018420 PMC5160927

[B107] Wang G. Ye C. Zhang J. Koziol L. Bever J. D. Li X. (2019). Asymmetric facilitation induced by inoculation with arbuscular mycorrhizal fungi leads to overyielding in maize/faba bean intercropping. J. Plant Interact. 14, 10–20. 10.1080/17429145.2018.1550218

[B108] Wang W. Shi J. Xie Q. Jiang Y. Yu N. Wang E. (2017). Nutrient exchange and regulation in arbuscular mycorrhizal symbiosis. Mol. Plant 10, 1147–1158. 10.1016/j.molp.2017.07.012 28782719

[B109] Waterman R. J. Klooster M. R. Hentrich H. Bidartondo M. I. Merckx V. (2013). Mycoheterotrophy: The Biology of Plants Living on Fungi. ed Merckx V. (New York, NY: Springer-Verlag).

[B110] Waters J. R. Borowicz V. A. (1994). Effect of clipping, benomyl, and genet on 14C transfer between mycorrhizal plants. Oikos 246–252. 10.2307/3546272

[B111] Weremijewicz J. O'Reilly L. S. L. Janos D. P. (2018). Arbuscular common mycorrhizal networks mediate intra-and interspecific interactions of two prairie grasses. Mycorrhiza 28, 71–83. 10.1007/s00572-017-0801-0 28986642

[B112] Weremijewicz J. Sternberg L. D. S. L. O. R. Janos D. P. (2016). Common mycorrhizal networks amplify competition by preferential mineral nutrient allocation to large host plants. New Phytol. 212, 461–471. 10.1111/nph.14041 27265515

[B113] Werner G. D. Kiers E. T. (2015). Partner selection in the mycorrhizal mutualism. New Phytol. 205, 1437–1442. 10.1111/nph.13113 25421912

[B114] Wilkinson D. M. (1998). The evolutionary ecology of mycorrhizal networks. Oikos 407–410. 10.2307/3546985

[B115] Wipf D. Krajinski F. van Tuinen D. Recorbet G. Courty P. E. (2019). Trading on the arbuscular mycorrhiza market: from arbuscules to common mycorrhizal networks. New Phytol. 223, 1127–1142. 10.1111/nph.15775 30843207

[B116] Wu B. Nara K. Hogetsu T. (2001). Can 14C-labeled photosynthetic products move between *Pinus densiflora* seedlings linked by ectomycorrhizal mycelia? New Phytol. 149, 137–146. 10.1046/j.1469-8137.2001.00010.x 33853229

[B117] Wu B. Nara K. Hogetsu T. (2005). Genetic structure of Cenococcum geophilum populations in primary successional volcanic deserts on Mount Fuji as revealed by microsatellite markers. New Phytol. 165, 285–293. 10.1111/j.1469-8137.2004.01221.x 15720640

[B118] Xiao Y. Li L. Zhang F. (2004). Effect of root contact on interspecific competition and N transfer between wheat and fababean using direct and indirect 15N techniques. Plant Soil 262, 45–54. 10.1023/B:PLSO.0000037019.34719.0d

[B119] Zhang H. Wang X. Gao Y. Sun B. (2020). Short-term N transfer from alfalfa to maize is dependent more on arbuscular mycorrhizal fungi than root exudates in N deficient soil. Plant Soil 446, 23–41. 10.1007/s11104-019-04333-1

[B120] Zimmer K. Meyer C. Gebauer G. (2008). The ectomycorrhizal specialist orchid *Corallorhiza trifida* is a partial myco-heterotroph. New Phytol. 178, 395–400. 10.1111/j.1469-8137.2007.02362.x 18221248

