# Supplementary material for: Common Mycorrhizae Network: A Review of the Theories and Mechanisms Behind Underground Interactions
Source: Front Fungal Biol. 2021 Sep 30;2:735299. doi: 10.3389/ffunb.2021.735299 (PMC10512311; doi:10.3389/ffunb.2021.735299)
Supplement: Supplementary file 1 [file Table_1.DOCX]

Supplementary Material

Table 1: Summary of some previous paper dealing with N transfer between plants connected by a common mycorrhiza network (CMN).

| **Paper** | **Experiment type** | **Direction** | **Type of inoculum** | **Prove direct path?** | **Transfer proportion/Outcomes** |
| --- | --- | --- | --- | --- | --- |
| Xiao et al., (2004) | Pot experiment – with solid barrier vs nylon mesh barrier 30-μm vs no barrier | *Fababean* → wheat | Soil Born (not specified) | No, flow of solution can occur between compartments | N transferred from *fababean* to wheat was 7.4 mg.pot^−1^ |
| He et al., (2005) | Field – with no mesh barrier | EM →EM/AM/dual | Soil born (not specified) | No, indirect pathway may still occur | Little N had been translocated from donors to woody species. Both EM and AM plants in close proximity to labeled plant acquired ^15^N |
| Meding & Zasoski, (2008) | Pot experiment - with 30µm mesh + 0.5 cm air gap | (1) forb → forb  (2) forb → grass  (3) grass → forb  (4) grass → grass or forb/grass mix → forb/grass mix | Soil born (not specified) | No, indirect pathway may still occur | Between 0.03% forb to forb and 0.15% mix to mix transfer of ^15^N. Transfer was greater when the grass was the receiver. |
| Meng et al., (2015) | Solid barrier, vs nylon mesh barrier 30-μm vs no barrier | Legume (+AM/+R or +AM/-R) → Maize | AM inoculum (non-sterile): (*Bradyrhizobium japonicum* and *Glomus mosseae* ) | No, flow of solution can occur between compartments | N transfer accounted for 3.13–6.01% of the N uptake of maize. N was transferred under non-inoculation conditions too. |

| **Paper** | **Experiment type** | **Direction** | **Type of inoculum** | **Prove direct path?** | **Transfer proportion/Outcomes** |
| --- | --- | --- | --- | --- | --- |
| Weremijewicz et al., (2016) | Greenhouse – with Cone-teiner with 40µm nylon mesh membrane + Gore Tex – Cone-teiners rotation *vs* no rotation *vs* close cone-teiners | Individuals of *Andropogon gerardii* | Several species of AM fungi | No, indirect pathway may still occur | Receiver non-shaded plants received the greatest amount of ^15^N. Intact CMNs, obtained at least 2.8-fold more ^15^N. |
| Wang et al., (2016) | Greenhouse/growth chamber – with 30µm mesh + 0.5 cm air gap | Legume (+AM/+R or +AM/-R) → Maize | 500 spores into the soil close to the roots (and 2 mL rhizobium solution for the +R treatment) | No, indirect pathway may still occur | The percentage N in maize derived from transfer (%NDFT) increased from 11.1% with only AM fungal inoculation to 15.2% with AM+Rhizobia. |
| He et al., (2019) | Microcosm experiment – with 20.0-μm nylon mesh (M+ treatment), 0.45-μm nylon mesh (M− treatment) + air gap | *Cinnamomum camphora* (donor) → *C. camphora*, *Broussonetia.papyrifera*, and *Bidens pilosa* | AM *Glomus etunicatum* - root pieces, hyphae, and ~ 100 spores per gram inoculum | No, indirect pathway may still occur | Bigger plants acquired more ^15^N. Heterospecific individuals acquired more ^15^N than the conspecific. %N transfer ranged from 0.09% to 0.22%. |
| Wahbi et al., (2016) | Greenhouse - without any mesh barrier | Central legume (*Vicia faba*) donor (nodulated) surrounded either by 10 other legume or by wheat | *Rizophagus irregularis* - 1000 (AM1) and 2000 (AM2). | No, indirect pathway may still occur | N transferred from *V. faba* to wheat were higher in AM1 (50%) and AM2 (32%) treatments compared with that of the uninoculated treatment (15%). |

| **Paper** | **Experiment type** | **Direction** | **Type of inoculum** | **Prove direct path?** | **Transfer proportion/Outcomes** |
| --- | --- | --- | --- | --- | --- |
| Zhang et al., (2020) | Field (intercropping) – without any mesh barrier | alfalfa (*Medicago sativa*) → Maize | Soil born (not specified) | No, indirect pathway may still occur. | N transfer rate ranged from 8% to 11% in 2015 and from 6% to 9% in 2016. N fixation was highly positively correlated with N transfer. |
| Fernandez et al., (2020) | Outdoor pots – with sole grown *vs* nylon (Nitex®) cloth with 1 μm *vs* 30 μm nylon *vs* no mesh | One-year-old bare-root oak seedlings (*Quercus petraea* (Matt.) Liebl.) → grass tufts (*Molinia caerulea*) **or** oak seedling | Soil born (not specified) | No, indirect pathway may still occur. | Mesh had very small effect on ^15^N transport. ^15^N allocation to shoots was much higher in receiver *Molinia* than in receiver oak. |
| Fang et al., (2021) | Greenhouse – with 37 µm hardware cloth + air gap | White clover (+AM/+R or +AM/-R) → Citrus | *Rhizophagus intraradices* + rhizobium for the +R treatment | No, indirect pathway may still occur. | %N transfer accounted  for 1.42–1.71% of the citrus total N. The combined nodulation/  mycorrhization enhanced 27.2% of N transfer. |
